# Translation of Circular RNAs: Functions of Translated Products and Related Bioinformatics Approaches

**DOI:** 10.2174/1574893618666230505101059

**Published:** 2023-10-03

**Authors:** Jae Yeon Hwang, Tae Lim Kook, Sydney M. Paulus, Juw Won Park

**Affiliations:** 1 Department of Computer Science and Engineering, University of Louisville, Louisville, KY 40292, USA;; 2 KY INBRE Bioinformatics Core, University of Louisville, Louisville, KY 40292, USA;; 3 CIEHS Biostatistics and Informatics Facility Core, University of Louisville, Louisville, KY 40292, USA

**Keywords:** Circular RNA, translation of circRNA, backsplicing, internal ribosome entry site, high-throughput sequencing, bioinformatics, computational biology

## Abstract

Over the past two decades, studies have discovered a special form of alternative splicing (AS) that produces a circular form of RNA. This stands in contrast to normal AS, which produces a linear form of RNA. Although these circRNAs have garnered considerable attention in the scientific community for their biogenesis and functions, the focus of these studies has been on the regulatory role of circRNAs with the assumption that circRNAs are non-coding. As non-coding RNAs, they may regulate mRNA transcription, tumor initiation, and translation by sponging miRNAs and RNA-binding proteins (RBPs). In addition to these regulatory roles of circRNAs, however, recent studies have provided strong evidence for their translation. The translation of circRNAs is expected to have an important role in promoting cancer cell growth and activating molecular pathways related to cancer development. In some cases, the translation of circRNAs is shown to be efficiently driven by an internal ribosome entry site (IRES). The development of a computational tool for identifying and characterizing the translation of circRNAs using high-throughput sequencing and IRES increases identifiable proteins translated from circRNAs. In turn, it has a substantial impact on helping researchers understand the functional role of proteins derived from circRNAs. New web resources for aggregating, cataloging, and visualizing translational information of circRNAs derived from previous studies have been developed. In this paper, general concepts of circRNA, circRNA biogenesis, translation of circRNA, and existing circRNA tools and databases are summarized to provide new insight into circRNA studies.

## 1. INTRODUCTION

Circular RNAs (circRNAs) are covalently closed single-stranded RNAs, which are distinct from usual linear RNAs. The structure of the circRNA was first identified as viroids which, unlike viruses, are uncoated infectious RNAs pathogenic to certain plants [1]. Apart from those found in viroids, circular forms of RNA were also found in the cytoplasm of various eukaryotes, including archaea, plants, flies, mice, and humans [2, 3]. In both cases, the closed circular structure of circRNAs was confirmed by their slower electrophoretic mobilities and by electron microscopy [2, 4]. These cellular circRNAs are generated mainly by backsplicing events during the splicing of pre-mRNAs [5]. While viroid circRNA sequences are self-complementary and have base-pairing secondary structures [1], circRNAs found in eukaryotic cells mainly contain parts of transcribed mRNAs of the coding region, most commonly of exonic sequences.

With the advent of recent high-throughput sequencing techniques, more extensive studies on circRNAs have been conducted in the last ten years, and numerous circRNAs have been searched from genomic sequencing data, such as RNA-seq data [6].

It has been found that circRNAs are widely spread throughout organisms and abundantly expressed in cells. At first, circRNAs were considered to be by-products from noncanonical RNA splicing, and their biological roles were ignored; however, more biological functions have recently been identified by extended studies. One of the well-known functions of circRNAs is as microRNA (miRNA) sponges. With the antisense sequences to miRNAs, circRNAs absorb miRNAs and regulate the interaction between miRNAs and mRNAs. Similarly, some circRNAs interact with RNA-binding proteins (RBPs) and act as RBP sponges, perturbating the interactions of the RBPs. circRNAs can interact with other proteins, facilitate RNA- or DNA-protein interactions, and modify the transcription rate of their own parent genes [7].

Lacking 5’cap and the structures for canonical translation initiation, circRNA was classified as a type of non-coding RNA initially; however, since most circRNAs originate from coding regions, they have been believed to be translatable into proteins and take more direct roles. In fact, many cases of circRNA translation have been reported. circRNAs are found in most sub-compartments in the cell, but the majority of them are found in the cytoplasm. circRNA expressions are often tissue-, cell type-, or stage-specific and take various biological roles, affecting different stages of development and diseases, including cancer. Differential circRNA expression profiles depending on various conditions and their expression in blood and other peripheral tissues *via* endosomes and microvesicles make them excellent candidates as a biomarker.

With more available high-throughput sequencing data and the development of computational tools for identifying and characterizing the translation of circRNAs, researchers have come to better understand the functional role of proteins derived from circRNAs. In addition, new web resources for annotating, cataloging, and visualizing translational information of circRNAs derived from previous studies have been developed. Here, we discuss circRNA biogenesis, the direct functions of translated products of circRNAs, and existing bioinformatics tools and databases closely associated with the translation of circRNAs to provide new insights into circRNA studies.

## 2. circRNA BIOGENESIS

**Fig. (1).**
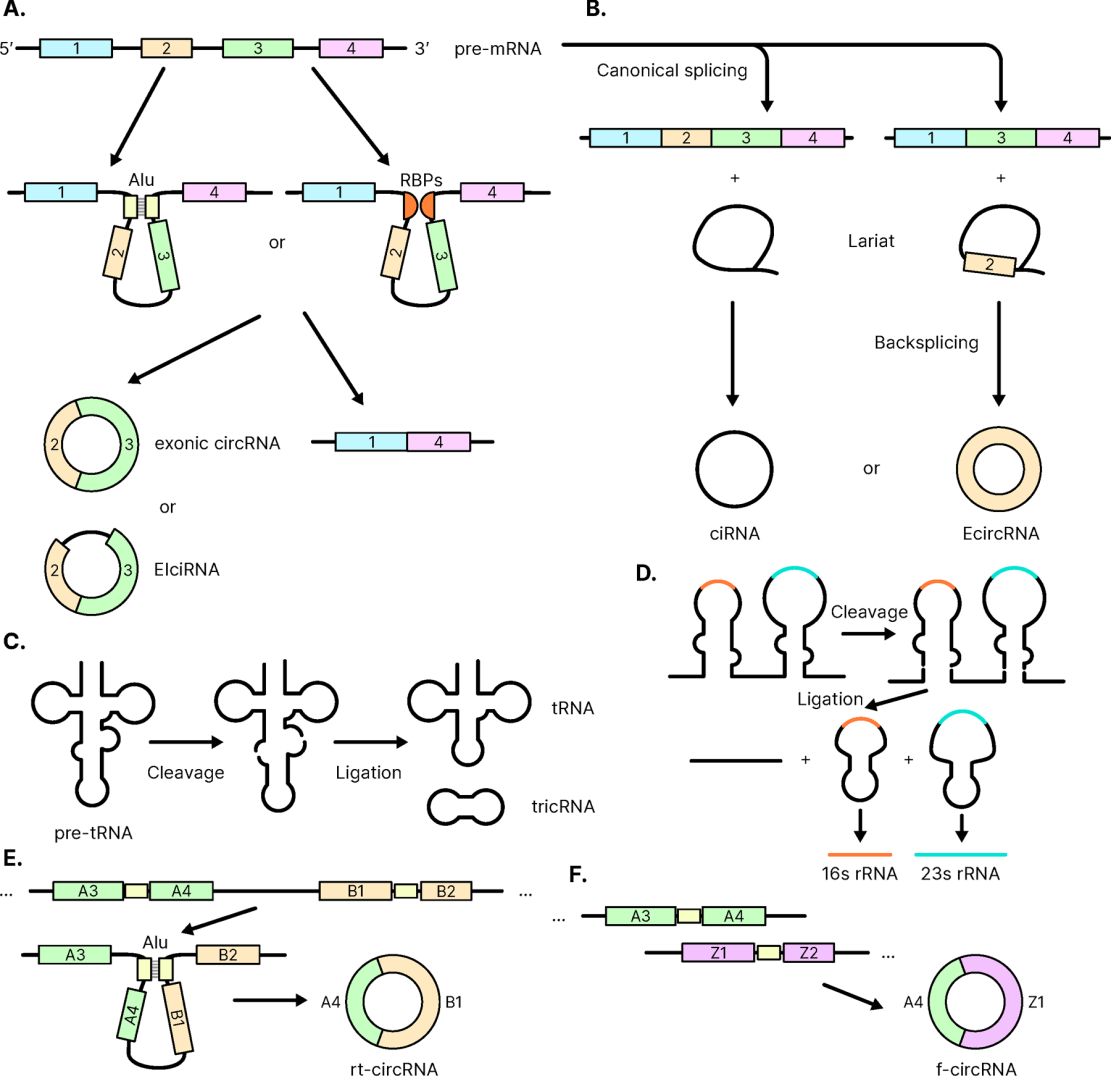
The biogenesis of circRNAs. (**A**). CircRNAs can be formed by backsplicing of pre-mRNA with assistance from reverse complement Alu sequences or RNA-binding proteins. (**B**). CircRNAs can also be formed from lariats. Circularized RNA can also arise as a byproduct of splicing (**C**). tRNA or (**D**). rRNA. (**E**). Read-through circRNAs (rt-circRNAs) are formed from exons from two nearby genes; however, their function is yet unclear [24]. (**F**). Fusion circRNAs (f-circRNAs) are made of exons from genes in different chromosomes and result from chromosomal translocations. f-circRNAs contribute to cellular transformation, increased tumorigenicity, and therapy resistance in cancer [16]. (*A higher resolution / colour version of this figure is available in the electronic copy of the article*).

**Fig. (2).**
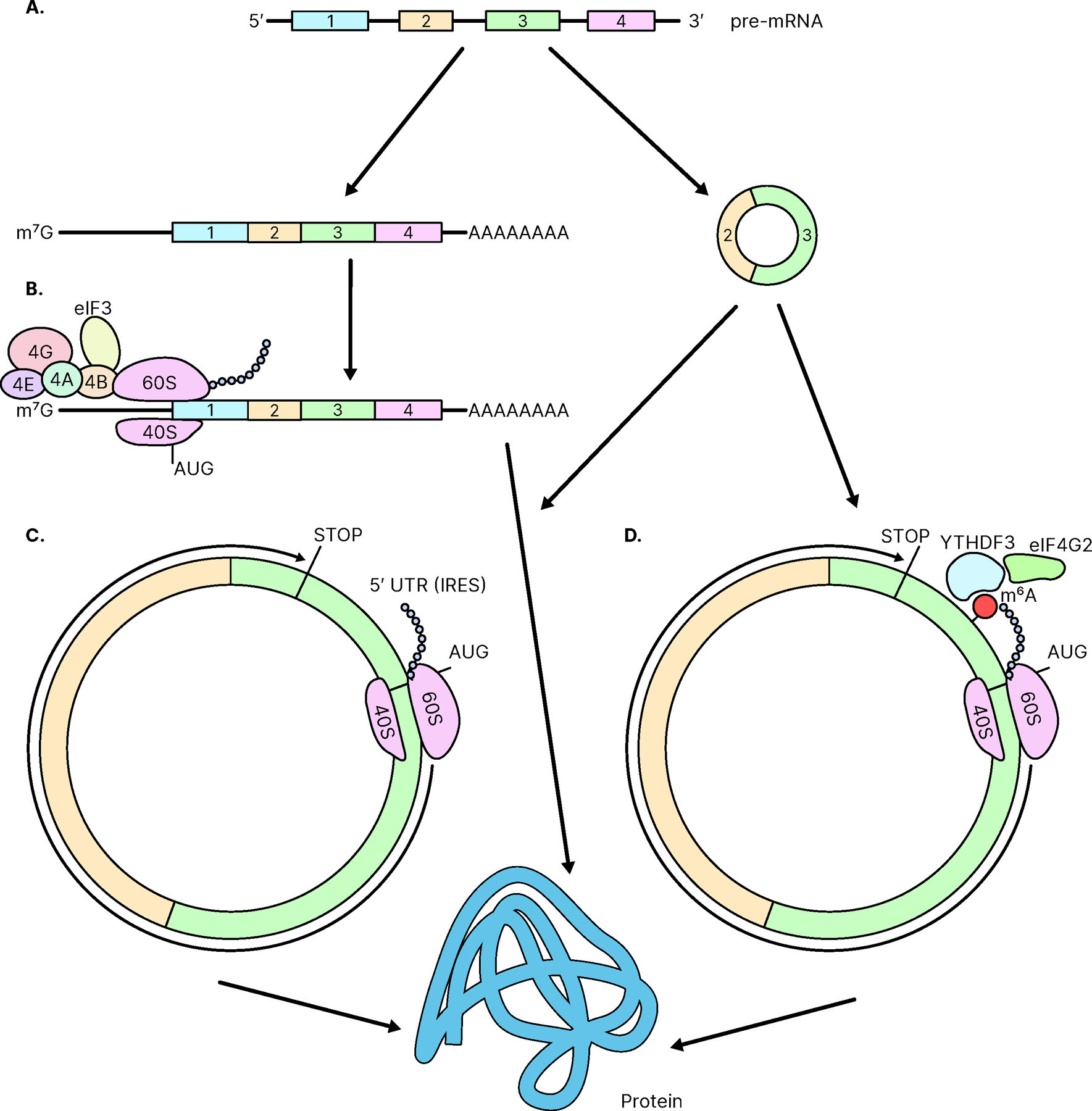
The translation of circRNAs. (**A**). pre-mRNA is spliced and may yield linear and circular RNA. (**B**). Cap-dependent translation of linear mRNA. (**C**). Cap-independent translation from an independent ribosome entry site (IRES). (**D**). Cap-independent translation from an m^6^A nucleotide. (*A higher resolution / colour version of this figure is available in the electronic copy of the article*).

circRNAs are abundant, conserved, and diverse [8]. Many different circRNAs are generated in eukaryotic cells depending on different circularization mechanisms. Back-splicing is the major mechanism that generates circRNAs. During splicing, an unconventional head-to-tail splicing occurs by covalently linking an upstream 5’ splicing acceptor site to a downstream 3’ splicing donor site, resulting in a circularized RNA closed at backsplicing junction (BSJ) (Figs. 1 and 2A) [8–10]. This non-canonical splicing event usually involves Alu repeats or RNA-binding proteins (RBPs) and yields circRNAs with only exonic sequences (EciRNAs) (Fig. 1A) or both exonic and intronic sequences (EIcircRNA) (Fig. 1B) depending on where the backsplicing event happens [8, 10, 11]. Lariat formation is another mechanism of generating circRNAs during splicing. Some intronic lariats, circularized at the branchpoint, are resistant to de-branching enzymes in the cell, thus allowing exoribonucleases to digest the linear part only up to the branch point and stabilize the structure, resulting in circular intronic RNAs (ciRNAs) (Fig. 1B) [12]. In addition, circRNA can be generated from tRNA (tricRNA) [13] and rRNA (Figs. 1C and D) [14]. In complex cases, a read-through circRNA (rt-circRNA) can be generated from exons of two nearby genes (Fig. 1E) [15], and a fusion circRNA (f-circRNA) can be made from exons of two different chromosomes *via* chromosomal translocations, and deletions (Fig. 1F) [16].

## 3. FUNCTION OF circRNA

Endogenous circRNAs are widely expressed in all human tissues, and some circRNAs are conserved in different species. circRNAs are expressed in various cell tissues, particularly in nervous tissue [17], and take roles in embryonic development, cell signaling, cell cycle, and stress responses. Tissue- or cell-type-specific differential expression profile of circRNAs are common among developmental stages and diseases, including cancer.

### 3.1. Interaction with Transcripts

circRNAs can interact with transcripts. One of the well-known roles of circRNAs is as miRNA sponges. With multiple miRNA binding sites in a stable circularized structure, circRNAs can sequester miRNA activity, consequently up-regulating miRNA target gene expression. Most circRNAs are observed to be located in the cytoplasm. For example, ciRS-7 was identified to be expressed abnormally in many types of cancer and to have more than 70 miR-7 target sequences that are not sliced by miR-7 [18]. CiRS-7 overexpression increased the expression levels of genes that miR-7 targets by suppressing miR-7 activity. Circ-Sry, a testis-specific circRNA derived from the sex-determining gene *Sry* of the Y chromosome in mice, acts as a miR-138 sponge affecting sex determination [19]. circ_Lrp6 is found in vascular smooth muscle cells and is known to regulate miRNA-145 function through sponging. circ_Lrp6 was found to essentially buffer the miRNA to sequester most of its function in the vascular smooth muscle cells. Similarly, circ-ITCH, found in gastric cancer cells, acts as a sponge of miR-199a-5p and, through this function, suppresses metastasis of gastric cancer. In addition to these cases, more evidence has been found that circRNAs play a role as miRNA sponges by using bioinformatic tools and high-throughput sequencing data. There are more regulatory roles of circRNA related to transcripts other than acting as miRNA sponges. For example, some cancer cells produce antisense circRNAs against tumor suppressor gene transcripts modulating their parental gene expression [20].

### 3.2. Interaction with Proteins

circRNAs can also interact with proteins. Similar to miRNA sponges, circRNAs can bind to RBPs and act as RBP sponges by sequestering RBPs from their targets. circMbl can interact with MBL protein and help maintain the expression level of both mbl and circMbl itself. It is also known that circEIF3J and circPAIP2 interact with RNA Pol II and affect the expression level of the parental genes. In other cases, circRNAs interact with transcription factors and regulate the transcription of some genes. Other functions of circRNAs interacting with RNAs or proteins include sequestering proteins from interacting with DNA or RNA, blocking protein-protein interactions, acting as chromatin remodelers, and increasing nuclear distribution or cytoplasmic distribution.

### 3.3. Direct Translation of circRNA

circRNAs can affect translation indirectly by inhibiting the translation of linear mRNAs by competing with standby ribosomes. On top of these regulatory roles, some circRNAs are actively translated in the cell and take more direct roles by synthesizing proteins. Some proteins translated from circRNAs function through the sites shared with their full-length counterpart mRNA, such as by competitively binding to some molecules. For example, YAP is a key component of the Hippo pathway, whose inhibition can promote apoptosis, suppress proliferation, and restrain metastasis of cancer cells. YAP can be negatively regulated by its circular RNA (circYap) through the suppression of the assembly of Yap translation initiation machinery. Overexpression of circYap in cancer cells significantly decreased YAP expression but did not affect its mRNA expression levels, thus remarkably suppressing proliferation, migration, and colony formation of the cells [21]. In other cases, circRNAs might gain their function from unique sequences in their C-termini translated beyond the splice junction. For instance, circ-E-cadherin acts as a template for a 254-aa protein with a unique 14-aa sequence at the C-terminus. This 14-aa sequence interacts with and activates EFGR, which subsequently activates STAT3 and enhances tumorigenicity in glioblastoma [22]. circFGFR1A, a circRNA transcribed from the FGFR1 gene that contains IRES, was also predicted to have strong translation activity. The encoded protein circFGFR1p functions as a negative regulator of FGFR1 by suppressing cell proliferation under stress conditions. Some cases of circRNA translation may produce rapidly degraded peptides that regulate immune surveillance rather than generating functional proteins [23].

## 4. TRANSLATION OF circRNA

Having a covalently closed circularized structure, circRNA is more resistant to exoribonucleases, such as RNase R, and has a longer half-life in the cell compared to its linear RNA counterpart. If circRNA is actively translated in the cell, having a circularized formation already, which is the functional formation when a linear mRNA is translated, it may have advantages as a more efficient transcript for protein synthesis. In addition, a circularized structure results in translation termination in the proximity of the start codon, thus accommodating the next round of translation more efficiently. This is done by recycling the ribosome, even theoretically providing an infinite translation loop as long as the template is not degraded. However, circRNAs are still missing some elements needed for translation.

Some circRNAs do not have coding sequences since they originate from an intron region. Even the majority of circRNAs that originate from exons only contain a part of the coding region and do not have a 3’poly(A)tail. More importantly, spliceosome-mediated backsplicing results in no 5’cap structure at the beginning of the circRNA sequence. Consequently, circRNAs are not translated into proteins by canonical cap-dependent initiation. In fact, circRNAs as viroids are not translated, rather having unique rod-like secondary structures from base-pairing, and they are replicated by RNA polymerase II in the host cell and function as ribozymes. Thus, even when cellular circRNAs were found, they were considered non-coding RNAs and ignored as byproducts of noncanonical RNA splicing. However, circRNAs are observed to be primarily located in the cytoplasm. The fact that circRNAs are more stable than linear RNAs in the cytoplasm constantly led researchers to think that they might be translated and that the covalently closed circular structure would serve to be more stable, reliable, and competitive gene expression material, even making infinite production with one transcript. It was not long before evidence of the translation of circRNA was found. There were experimental data supporting the potential translation of circRNAs reported both *in vitro* and *in vivo*.

The first natural circRNA found that encodes a protein is the genome of the hepatitis delta virus, not by direct translation from the circRNA, but by first making cDNA [25]. It was known that circRNA can be translated by a rolling circle amplification (RCA) in a cell-free *Escherichia coli* translation system; however, it was not shown that they are translatable in a eukaryotic cell translation system [26]. The first translation of circRNA was reported from a 220-nt long circRNA of the viroid-like satellite RNA related to the rice yellow mottle virus. An internal ribosome entry site (IRES) facilitated translation initiation, and polypeptides were produced from two or three overlapping open reading frames (ORFs) [27]. Later, the translation of circRNAs with an infinite ORF in eukaryotic systems was tested. Circular RNAs were translated in rabbit reticulocyte lysate without IRES, a poly(A)tail, or a 5’cap structure. It was also shown that the circular RNA is efficiently translated in living human cells [28]. circMbl3, which originates from a fly muscleblind locus, has been found to be translated into a polypeptide under stress conditions like starvation in fly head extracts. The protein isoform synthesized from circMbl3 was confirmed by mass spectrometry [29]. circ-ZNF609, which contains an ORF spanning from the start codon and stop codon, was found to be associated with heavy polysomes, and it is translated into a protein by a splicing-dependent and cap-independent manner and controls myoblast proliferation [30].

### 4.1. Translation Initiation Mechanism of circRNA

Since circRNAs are mainly generated from backsplicing during non-canonical RNA splicing events, circRNAs lack some elements needed to be actively translated into proteins. Unlike linear mRNAs, circRNAs have neither a 5’cap nor a 3’poly(A)tail. Translation of most linear mRNAs in eukaryotic cells is initiated by 5’cap-dependent translation. Translation initiation factor 4E (eIF4E) first recognizes the 5’ 7-methyl guanylate cap (m^7^G) of mRNA and binds to it (Fig. 2B). Other initiation factors bind to the 40 S ribosomal subunit and form an initiation complex by interacting with eIF4E which is bound to the 5’cap [31]. Then, the complex reads through the mRNA from 5’ to 3’ for a start codon to begin protein synthesis. On the other hand, the translation initiation of circRNAs is cap-independent. Suggested mechanisms of initiation of circRNA translation are through IRES [32] and through N6-methyladenosine (m^6^A) modification containing short sequences by directly binding initiation factors to circRNAs [28, 33–35]. Although cap-independent translation is not as efficient as cap-dependent translation, it has been suggested that exogenous circRNA for robust and stable protein expression in eukaryotic cells by cap-independent translation could be a promising alternative to linear mRNA [34].

### 4.2. Internal Ribosome Entry Site (IRES) Mediated Translation of circRNA

IRESs are located in the 5’-untranslated region (5’-UTR) and form secondary structures that interact with the ribosomes in the middle of mRNAs inducing translation initiation [36]. IRESs in eukaryotic mRNAs facilitate translation initiation under stress conditions, including nutrition starvation, apoptosis [37], mitosis [38] and tumorigenesis [39]. They provide an alternative way to continue translation when cap-dependent translation is suppressed as well as when it is fully active [40]. Cap-independent translation initiated by an IRES shows different efficiency depending on various conditions of cells and is generally less efficient than cap-dependent translation [41]. circRNA construction with IRES has shown that it can be translated into a protein (Fig. 2C) both *in vitro* and *in vivo* [32].

### 4.3. N6-methyladenosine (m^6^A) Motif Mediated Translation of circRNA

Methylation of the nitrogen at position 6 in the adenosine base on mRNA is a post-transcriptional modification found in many eukaryotic cells. The m^6^A modification is reversible and can be increased by m^6^A methyltransferase complex containing methyltransferase 3 (METTL3) as the SAM-binding subunit [42] or can be decreased by demethylase FTO [33]. m^6^A modification site in circRNA can facilitate translation initiation of circRNA with the reader YTHDF3 recruiting eIF4G2 to the m^6^A carrying circRNA (Fig. 2D) [33, 43]. A single m^6^A residue is sufficient for the initiation of translation, suggesting the role of circRNA-derived proteins in cellular responses to environmental stress [33]. For example, m^6^A modifications in circZNF609, circE7, and other circRNAs were shown to induce the translation of those circRNAs [30, 44]. In addition, many short sequences with m^6^A-induced ribosome engagement sites (MIRESs), including m^6^A sites, have been reported to function as IRES-like elements to drive circRNA translation [29, 30, 33]. It was shown that circRNAs in human cells could be efficiently translated using 19-nt short consensus sequences containing m^6^A motifs, such as RRm^6^ACH [33].

### 4.4. Experimental Validation of circRNA Translation

It has been shown that circRNAs can be translated into proteins both *in vitro* and *in vivo* [28, 29, 32, 35, 45]. However, it was not clear if they are actively translated into functional proteins endogenously [46]. While most circRNAs contain coding sequences that originate from one or more exons, they lack a 5’cap and a polyA tail, which are required for canonical translation initiation. Thus, if circRNAs are translated, the initiation of translation is cap-independent. The translation of circRNA requires the formation of a ribosome-circRNA complex; however, ribosome association does not necessarily mean translation. For instance, both circPABPN1 and circFAM120A interact with the ribosome, but they are not translated; instead, they function by regulating their cognate mRNA translation [47–49].

Cap-independent initiation of translation from circRNA by either IRES or m^6^A site has been reported in human cells. Yang *et al*. have shown that a circRNA, circ-FBXW7A, encodes a novel protein, FBXW7–185aa, which contributes to the inhibition of glioma tumorigenesis (Table 1) [50–75]. Begum *et al*. has displayed a novel tumor-suppressive protein, SHPRH-146aa, which is encoded by circ-SHPRH [68]. Another tumor suppressive protein, PINT87aa, which is encoded by circPINTexon2, has been identified in glioblastoma [50].

Yin *et al*. showed that circFAM188B promotes proliferation while inhibiting the differentiation of chicken skeletal muscle satellite cells (SMSCs). circFAM188B encodes and translates into circFAM188B-103aa. In the study, IRES in circRAM188B supported the coding potential, and peptides of circFAM188B-103aa were detected by both western blot assay and LC-MS/MS analysis. The researchers further verified that the role of circFAM188B-103aa in chicken myogenesis is consistent with that of its parent transcript circFAM188B [69]. More reported cases of translated proteins from various circRNAs are listed in Table 1.

## 5. BIOINFORMATICS TOOLS TO SEARCH TRANSLATION OF circRNA

With the advent of various high-throughput sequencing techniques, research on circRNA also has been extended, and genome-wide profiling of circRNA has been available recently. More than 40 bioinformatics tools have been developed for circRNA research, aiming for identification, annotation building databases, network analysis, prediction of coding ability, and so on [70]. Among those, some are more closely associated with the translation of circRNAs. CircPro [71], CircCode [72], CircPrimer 2.0 [73], and Rcirc [74] can identify circRNAs with coding potential as well as detect circRNAs using high-throughput sequencing data. WebCircRNA [75] can classify the circular RNA potential for coding and non-coding RNA using a machine learning-based method. MStoCIRC is an analysis tool to predict the translation capacity of circRNAs based on mass spectrometry data [76]. And CircRNADb provides human circRNAs with protein-coding annotations (Table 2) [77].

### 5.1. Identification and Annotation of circRNA Using High-throughput Sequencing Data

Those tools for identification utilize various alignment tools such as BWA, Bowtie, and STAR aligners with some exceptions; however, the core algorithm involves the detection of BSJ reads, which are similar to chimeric junction reads that are often found in gene fusion events. Any RNA-seq data and similar types of sequencing data, such as ribosome profiling data or Ribo-seq, CLIP-seq, ISO-seq, and miRNA-seq data, can be used for circRNA identification. Reducing false positives while identifying circRNAs from sequencing data is challenging. True BSJ reads it can be detected more efficiently by using rRNA-depleted paired-end RNA-seq data with over 100-bp read length in general to get sufficient read length containing the BSJ site. Single-end sequencing data can detect as many BSJ reads as paired-end sequencing data, but they are missing the information on flanking sequence next to the BSJ sites. In addition, RNase R-treated samples can be used for better identification of circRNAs, but some circRNAs sensitive to RNase R can be lost during sample library preparation. False-positive can be filtered out by using some assisting algorithms while detecting circRNAs. For example, CIRI filters false positives using GT-AG splicing signals as well as paired-end mapping information [78], and PredcircRNA implements machine-learning approaches factoring sequence composition, ALU and tandem repeat sequences, *etc*. [79].

### 5.2. Bioinformatics Tools to Identify Translation of circRNA

Emerging roles of direct translation from circRNAs are getting more attention from researchers. Some bioinformatics tools facilitate the investigation of circRNA translation, including identifying circRNA-derived proteins and their novel biological functions. Those tools require both RNA-seq and Ribo-seq data for better assessment of potential protein-coding circRNAs.

CircPro identifies potential protein-coding circRNAs using RNA- and Ribo-seq data, as well as predictions of ORFs from CPC, a support vector machine-based tool to assess the protein-coding potential using sequence features. CircPro first utilizes CIRI2 for de novo detection of circRNAs, then CPC for protein-coding potential and information of ORF. On real sequencing data, CircPro identified 6 out of 10 protein-coding circRNAs experimentally, which were verified by an earlier study [71]. Rcirc is a user-friendly package based on R language with lots of highly automatic functions for identifying the coding ability of circRNA and visualizing the feature in various aspects. Rcirc can be used to predict the de novo circRNAs based on RNA-seq data by calling CIRI2 and to identify their coding ability based on Ribo-seq data. Further, circRNAs of interest can be visualized using browser tools, such as IGV [80]. CircCode is another powerful tool for identifying circRNA coding ability. CircCode also predicts the coding ability of circRNAs using Ribo-seq data by detecting reads aligned to BSJ sites. CircCode attempt to improve CircPro using a different tool called BAS-iNET [81], an RNA classifier based on machine learning methods for coding and non-coding RNA identification [71]. CircPro uses CPC, which was originally used to calculate potential coding scores of linear mRNAs, and assumes that the start codon is at the start position of translation. Thus, it may leave out some actually translated circRNAs since some cases of circRNA translation do not use the start codon [71]. The authors claimed that using ribosome profiling data downloaded from NCBI, they found 3,610 and 1,569 translated circRNAs in humans and *A. thaliana*, respectively. CircPrimer 2.0 can also predict the translation potential of circRNAs. CircPrimer 2.0 predicts small ORFs in a cap-independent manner, as well as IRES, and m^6^A sites, which are time-consuming and labor-intensive to identify using experimental methods [73]. CircPrimer 2.0 also supports showing conserved circRNAs between humans and mice.

## 6. DISCUSSION AND FUTURE PERSPECTIVES

The intrinsic reason why the study of the translation of circRNAs is difficult is that there is only little portion of reads that covers backsplicing regions from high-throughput sequencing data such as Ribo-seq. However, as more evidence of circRNA translation accumulates, the more biological roles of proteins derived from circRNAs are being identified. Recent progress in sequencing techniques, mainly RNA-seq and Ribo-seq, provide more efficient methods for circRNA translation research. Furthermore, the recent development of bioinformatics tools for the identification and annotation of circRNAs expedites their study, which can be very time-consuming and labor-intensive if done by conventional experimental methods. Functions of protein products derived from circRNA are more direct compared to transcriptional functions and interactions with other proteins. Recent studies show that circRNA-derived peptides affect many diseases, including cancer.

Bioinformatics tools have identified many translatable circRNAs using both RNA-seq and Ribo-seq data. When identifying translatable circRNAs using Ribo-seq data, additional processing steps are required compared to RNA-seq data processing by trimming the linker sequence and filtering low-quality fragments and short fragments with multiple alignments to the reference. In addition, it is recommended to remove the tRNA and rRNA sequences that were introduced during Ribo-seq sample library preparation, which are unavoidable contaminations during flanking mRNA degradation up to the boundaries of the ribosome using exonucleases, such as RNase I. Since the length of the reads obtained by the Ribo-seq is usually less than 40 bp, it is even harder to find reads that span over BSJs. The difficulty really lies in detecting spanning reads that cover enough sequences that are uniquely identifiable but also have enough length to go around both sides of the BSJs. Still, the validation and quantification of their translation are further needed. Since circRNA expression is tissue-, cell-type-, and stage-specific, more accurate evidence of translation of circRNAs can be obtained when coupled RNA-seq, specifically rRNA-depleted RNA-seq data, and Ribo-seq data with good read depths are available.

## CONCLUSION

With a stable circularized structure, circRNA has the potential to serve as specialized templates for translation and for taking endogenous regulatory roles in eukaryotic cells. As more circRNA-derived peptides related to diseases are identified, the possibility that circRNAs can be good markers or targets for disease treatment becomes apparent. circRNAs also have the potential to serve as efficient vectors for translation; however, the mechanism of circRNA requires more elucidation.

Currently, available bioinformatics tools for circRNA study are mainly for identification by focusing on BSJ detection capturing BSJ spanning reads from high-throughput sequencing data, but there are not many tools for detecting translation of circRNAs yet. Some of the tools came up with detecting coding potentials by identifying the presence of IRES or m^6^A sites in the circRNA sequences or by directly detecting footprints of translating ribosomes on BSJ-spanning regions. With the addition of databases on protein products derived from the translation of circRNAs, bioinformatics approaches will reveal and validate more direct functions of the translation of circRNA.

## Figures and Tables

**Table 1.** Previously identified products from translation of circRNAs

Name	Size(nt)	Origin (gene: exon)	IRES or m⁶A	Translated products	Function (mRNA level)	Function (protein level)
circE7 [44]	472	HPV16 E7: exon 6–7,1	m⁶A	98 amino acid, E7 oncoprotein	Biomarker for presence of HPV’s, promote viral replication/host cell transformation	
circHER2 [58]	676	ERBB2: exon 3–7	IRES	1–103aa peptide HER2–103	Her2-103 deprivation attenuated cell proliferation in TNBC cancer cell line	
circALTO2 [59]	940	ALTO:ALTO ORF+ canonical splice site	m⁶A	248aa ALTO2 protein	Function remains unclear	
circPINTexon2 [50]	1084	Linc-Pint: exon 2	IRES	87-aa peptide PINT87 protein	Functions in glioma	
circPPP1R12A [60]	1138	PPP1A12A: exon 24,25	IRES	circPPP1R12A-73 aa small peptide	Promotes proliferation, migration and invasion of colon cancer cell line	
circDIDO1 [61]	1787	DIDO1:exin 2–6	IRES/m⁶A	1–529aa DIDO1–529 aa protein	Gastric cancer tumor suppressor, prognostic biomaker	
circSHPRH [62]	440	SHPRH: exon 26–29	IRES	SHPRH-146 aa	Human glioblastoma tumor suppressor	
circMAPK1 [63]	490	MAPK1: exon 2–4	IRES	MAPK1–109 aa short peptide	Vascular smooth muscle cells- simulatory- miR-22-3p sponge	Gastric cancer- inhibitory- new isoform by IRES-driven translation
circAKT3 [64]	524	AKT3: exon 3–7	IRES	AKT3–174 aa	miR-206, miR-516b-5p, miR-198, miR-515–5p, miR-17–5p, miR-335, and miR-144-p sponge	Glioblastoma-tumor suppressor - new isoform by IRES-driven translation
circAβ-a [65]	524	APP: exon 14–17	IRES	‘Aβ175’ Aβ-related protein	Possible role in pathology of Alzheimer’s disease	
circFNDC3B [66]	526	FNDC3B: exon 5,6	IRES	circFNDC3B-218 aa protein isoform	Interacts with IGF2BP3 and promotes the migration and invasion of gastric cancer	Colon cancer-tumor suppressor - new isoform by IRES-driven translation
circFBXW7 [48]	620	FBXW7: exon 3,4	IRES	FBXW7–185aa	Encodes for the novel protein	Induce cell cycle arrest + reduce proliferation in glioma cells
circSMO [67]	727	SMO: exon 3–6	IRES	SMO-193a.a new protein	Encodes for the novel protein	CSC maintenance, HH signaling in GBM
circNLGN [68]	813	NLGN: exon 2	N/A	circ164aa; NLGN173	Promotes pathological cardiac fibrosis	
circZNF609 [30]	874	ZNF609: exon 2	IRES/m⁶A	circ-ZNF609-encoded protein	Colorectal cancer-tumor suppressor-p53 upregulation, coronary artery disease inhibitory/anti-inflammatory potential miRNA sponge	Myogenesis-stimulatory - multiple isoforms from different ORFs by IRES-driven translation
circβ-catenin [69]	1129	β-catenin: exon 2–7	IRES	β-catenin-370 aa	Serves as decoy to prevent protein binding	Effective competitor
Circ-E-Cad [23]	733	CDH1: exon 7–10	IRES	254aa protein C-E-Cad	Prognostic factor in GBM	
circAXIN1 [70]	959	AXIN1: exon 2	IRES	AXIN1–295aa	Encodes for the novel protein	Oncogneic protein, promotes tumorigenesis
circPLCE1 [71]	1570	PLCE1: exon 2	IRES	411 amino acid protein circPlce1-411	Proliferation and migration of CRC cell lines	
circARHGAP35 [72]	3867	ARHGAP35: exon 2,3	m⁶A	large protein circARHGAP35 protein	Oncogenetic	Oncogenetic, promotes cancer cell progression
circEGFR [73]	249	EGFR:exon 14,15	IRES	rolling-translated EGFR rtEGFR	Glioma tumor suppressor - miR-183–5p sponge	Glioblastoma oncoprotein - new isoform by infinite rolling circle translation
circALTO1 [59]	762	ALTO: ALTO ORF	m⁶A	ALTO1 protein isoform	Function remains unclear	
circCHEK1 [74]	738	CHEK1: exon2-7	IRES	circCHEK1–246 aa	Evoke MM CIN	Increased MM CIN and osteoclast differentiation
circ-LINCPINT [75]	1084	LINC-PINT: exon 2	IRES	87-aa peptide: PINT87aa	Inhibits proliferation of glioma cells	

**Table 2.** Bioinformatics tools associated with the analysis of circRNA translation.

Name	Application	Platform	Required Tools	Published
CircPro [71]	Annotation with protein-coding potential	Unix/Linux	Perl	2017
CircRNADb [77]	Database for human circRNA with protein-coding annotations	Web-based	-	2016
CircPrimer 2.0 [73]	Annotation with protein-coding potential	Windows, Unix/Linux	Java	2022
Rcirc [74]	Identification of circRNA coding ability and visualization	Unix/Linux	R	2020
MStoCIRC [76]	Prediction of translatable circRNAs from MS/MS data	Windows, Unix/Linux	Python, R	2022
CircCode [72]	Identification of circRNA coding ability	Unix/Linux	Python, R	2019
WebCircRNA [75]	Webserver to assess the protein-coding potential	Web-based, Unix/Linux	-	2018

## LIST OF ABBREVIATIONS

BSJ: Backsplicing Junction
ciRNAs: Circular Intronic RNAs
EciRNA: Exonic circRNA
f-circRNA: fusion circRNA
RBPS: RNA-binding Proteins
rt-circRNA: read-through circRNA
